# Supplementary material for: Evolutionary sequence and structural basis for the distinct conformational landscapes of Tyr and Ser/Thr kinases
Source: bioRxiv. 2024 May 2:2024.03.08.584161. Originally published 2024 Mar 12. Preprint. [Version 2] doi: 10.1101/2024.03.08.584161 (PMC10979876; doi:10.1101/2024.03.08.584161)
Supplement: Supplement 1 [file media-1.pdf]

Supplementary information:

Evolutionary sequence and structural basis for the distinct conformational landscapes of Tyr and Ser/Thr kinases

Joan Gizzio[a,b], Abhishek Thakur[a,b], Allan Haldane[a,c], Carol Beth Post[d], Ronald M. Levy[a,b,1]

[a]Center for Biophysics and Computational Biology, Temple University, Philadelphia, Pennsylvania 19122

[b]Department of Chemistry, Temple University, Philadelphia, Pennsylvania 19122

[c]Department of Physics, Temple University, Philadelphia, Pennsylvania 19122

[d]Borch Department of Medicinal Chemistry and Molecular Pharmacology, Purdue University, West Lafayette, Indiana 47907

[1]To whom correspondence should be addressed.

Email: ronlevy@temple.edu

Competing Interest Statement: The authors declare no competing interests.

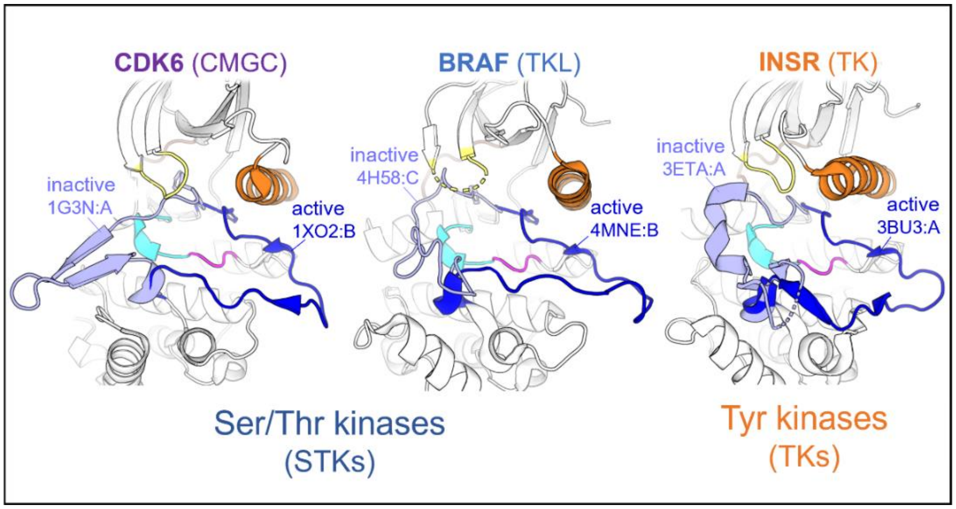

Figure S1. Overview of the three kinase targets and their active and inactive states, analyzed by Potts threading and molecular dynamics free energy simulations in this work.

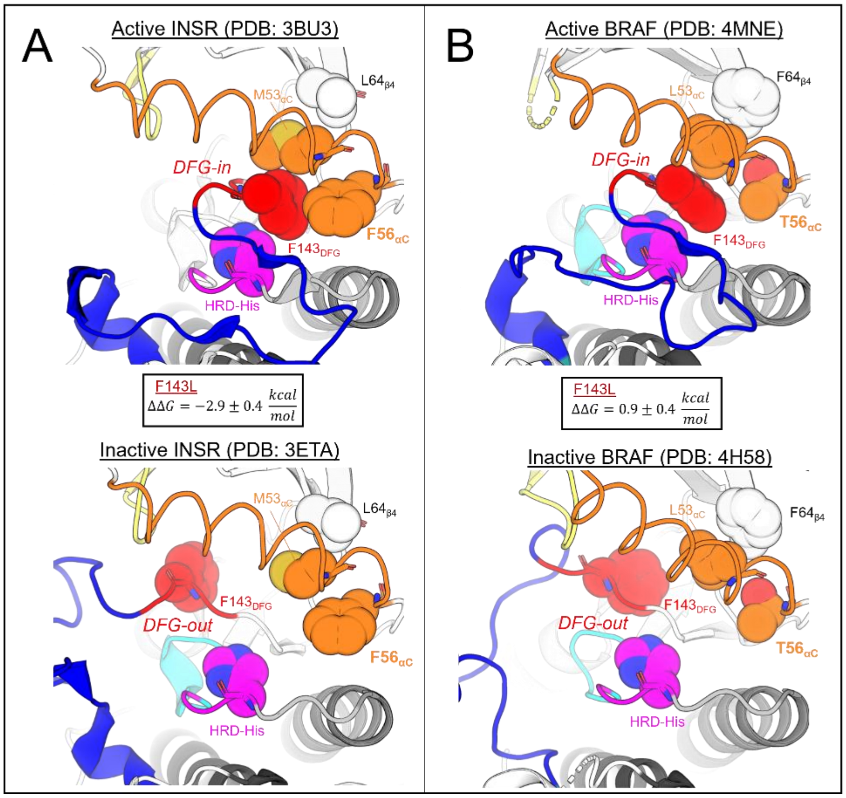

Figure S2. Potts threaded energies predict F143L to destabilize the R-spine of INSR, but not BRAF, consistent with FEP simulations (see Table S3 for data). The R-spine residue positions are labeled HRD-His, 143_{DFG}, $53_{\alpha C}$ and $64_{\beta 4}$. Residue $56_{\alpha C}$ is located next to the R-spine in the αC-helix. $\Delta\Delta G_{reorg}$ for the $F143L_{DFG}$ mutation is reported in the inset. Potts threaded energies suggest that interactions between $F143_{DFG}$ and $56_{\alpha C}$ are weaker in BRAF compared with INSR. Consequently, the $F143L_{DFG}$ mutation penalizes the active-state stability of the INSR R-spine, in contrast to BRAF where the mutational effect has an opposite sign. (A) Active DFG-in vs inactive DFG-out conformation of INSR. (B) Active DFG-in vs inactive DFG-out conformation for BRAF.

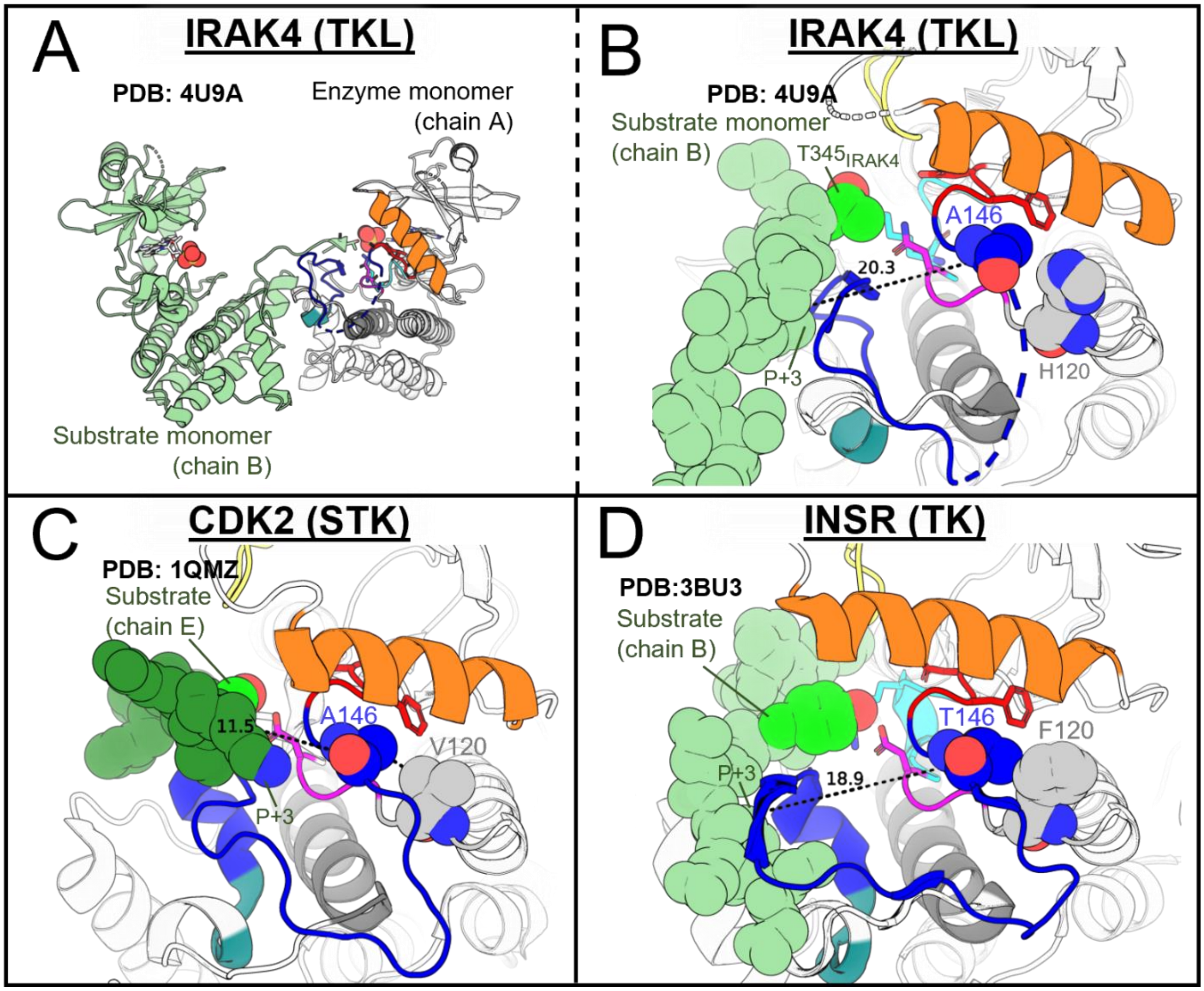

Figure S3. (**A**) Overview of the IRAK4 trans-phosphorylation dimer structure, PDB: 4U97. (**B**) Viewing the IRAK4 enzyme monomer and substrate activation loop from the opposing chain. Chain A represents the active enzyme monomer while a Thr residue (T345) from the activation loop of chain B serves as the substrate/phosphoacceptor. (**C**) CDK2 bound to a substrate peptide (PDB: 1QMZ) with the peptide oriented into the kinase cleft between the N-lobe and C-lobe (see Figure 6 for schematic). The peptide is bound nearby the DFG+2 residue (A146) in the activation loop N-terminal anchor. Relative to CDK2 (an STK), IRAK4 has an unstable N-terminal anchor according to the Potts model due to H120$_{HRD-2}$ (H307 in IRAK numbering), similar to the effect of F120 in TKs (e.g. INSR, see Figure 3). (**D**) INSR bound to a substrate peptide. INSR and many other TKs have a bulky aromatic residue at this position (F120) which has destabilizing effects on the activation loop N-terminus, consistent with evolutionary "decommissioning" of the cleft-oriented binding mode in TKs. Our Potts model calculations in Table S1 might suggest that H120 is present in IRAK4 for a similar reason – the STK-preferred "cleft" binding mode might be decommissioned in IRAK4, and possibly other TKLs, consistent with the substrate-bound structure in *A-B* and the nature of TKLs as "evolutionary intermediates" along the evolutionary path between STKs and TKs (Figure 2).

Table S1. Potts model results for each of the three kinases from Figure S3. Smaller values of ΔT reflect destabilization of the extended/active conformation of the activation loop relative to the inactive conformation. Mutating the STK-prevalent V120 in CDK2 to the residue seen in IRAK4 (V120H) destabilizes N-terminal anchor according to the Potts model ($\Delta\Delta T \approx -1$), and the reverse mutation in IRAK4 (H120V) recovers some stability of the N-terminal anchor ($\Delta\Delta T \approx +1$) to make the kinase more "STK-like". This is consistent with the observation in Figure S2B that IRAK4 utilizes the TK-preferred peptide binding mode, where the peptide (in this case, the activation loop of a substrate monomer) is bound far away from the enzyme's activation loop N-terminus.

Class	Kinase	HRD-2 (pos. 120)	Wildtype Potts ΔT	Mutation	ΔΔT
STK	CDK2	Val	5.3	V120H	-1.2
TK	INSR	Phe	0.5	F120H	0.1
TKL	IRAK4	His	2.5	H120V	0.89

Table S2. Alignment to PDB numbering conversion table.

Alignment	CDK6 (1G3N:A)	BRAF (4H58:C)	INSR (3ETA:A)
1	-	-	-
2	Q12	Q455	K995
3	Y13	I456	I996
4	E14	T457	T997
5	C15	V458	L998
6	V16	G459	L999
7	A17	Q460	R1000
8	E18	R461	E1001
9	I19	I462	L1002
10	G20	G463	G1003
11	E21	S464	Q1004
12	G22	G465	G1005
13	A23	S466	S1006
14	Y24	F467	F1007
15	G25	G468	G1008
16	K26	T469	M1009
17	V27	V470	V1010
18	F28	Y471	Y1011
19	K29	K472	E1012
20	A30	G473	G1013
21	R31	K474	N1014
22	D32	W475	A1015

Alignment	CDK6 (1G3N:A)	BRAF (4H58:C)	INSR (3ETA:A)
23	L33	-	R1016
24	N35	-	E1022
25	G36	-	A1023
26	G37	H476	E1024
27	R38	G477	T1025
28	F39	D478	R1026
29	V40	V479	V1027
30	A41	A480	A1028
31	L42	V481	V1029
32	K43	K482	K1030
33	R44	M483	T1031
34	V45	L484	V1032
35	R46	N485	N1033
36	V47	V486	E1034
37	Q48	T487	S1035
38	T49	A488	A1036
39	G50	P489	S1037
40	E52	P491	L1038
41	G53	Q492	R1039
42	M54	Q493	E1040
43	P55	L494	R1041
44	L56	Q495	I1042
45	S57	A496	E1043
46	T58	F497	F1044
47	I59	K498	L1045
48	R60	N499	N1046
49	E61	E500	E1047
50	V62	V501	A1048
51	A63	G502	S1049
52	V64	V503	V1050
53	L65	L504	M1051
54	R66	R505	K1052
55	H67	K506	G1053
56	L68	T507	F1054
57	E72	R508	T1055
58	H73	H509	C1056
59	P74	V510	H1057
60	N75	N511	H1058
61	V76	I512	V1059
62	V77	L513	V1060
63	R78	L514	R1061
64	L79	F515	L1062

Alignment	CDK6 (1G3N:A)	BRAF (4H58:C)	INSR (3ETA:A)
65	F80	M516	L1063
66	D81	G517	G1064
67	V82	Y518	V1065
68	C83	S519	V1066
69	T84	T520	S1067
70	V85	K521	K1068
71	S86	P522	G1069
72	R87	-	Q1070
73	K93	Q523	P1071
74	L94	L524	T1072
75	T95	A525	L1073
76	L96	I526	V1074
77	V97	V527	V1075
78	F98	T528	M1076
79	E99	Q529	E1077
80	H100	W530	L1078
81	V101	C531	M1079
82	D102	E532	A1080
83	-	G533	H1081
84	Q103	S534	G1082
85	D104	S535	D1083
86	L105	L536	L1084
87	T106	Y537	K1085
88	T107	H538	S1086
89	Y108	H539	Y1087
90	L109	L540	L1088
91	D110	H541	R1089
92	K111	I542	S1090
93	V112	I543	L1091
94	P115	T545	P1102
95	G116	K546	P1103
96	V117	F547	P1104
97	P118	E548	T1105
98	T119	M549	L1106
99	E120	I550	Q1107
100	T121	K551	E1108
101	I122	L552	M1109
102	K123	I553	I1110
103	D124	D554	Q1111
104	M125	I555	M1112
105	M126	A556	A1113
106	F127	R557	A1114

Alignment	CDK6 (1G3N:A)	BRAF (4H58:C)	INSR (3ETA:A)
107	Q128	Q558	E1115
108	L129	T559	I1116
109	L130	A560	A1117
110	R131	Q561	D1118
111	G132	G562	G1119
112	L133	M563	M1120
113	D134	D564	A1121
114	F135	Y565	Y1122
115	L136	L566	L1123
116	H137	H567	N1124
117	S138	A568	A1125
118	H139	K569	K1126
119	R140	S570	K1127
120	V141	I571	F1128
121	V142	I572	V1129
122	H143	H573	H1130
123	R144	R574	R1131
124	D145	D575	D1132
125	L146	L576	L1133
126	K147	K577	A1134
127	P148	S578	A1135
128	Q149	N579	R1136
129	N150	N580	N1137
130	I151	I581	C1138
131	L152	F582	M1139
132	V153	L583	V1140
133	T154	H584	A1141
134	S155	E585	H1142
135	S156	D586	D1143
136	G157	L587	F1144
137	Q158	T588	T1145
138	I159	V589	V1146
139	K160	K590	K1147
140	L161	I591	I1148
141	A162	G592	G1149
142	D163	D593	D1150
143	F164	F594	F1151
144	G165	G595	G1152
145	L166	L596	M1153
146	A167	A597	T1154
147	R168	T598	R1155
148	I169	V599	D1156

Alignment	CDK6 (1G3N:A)	BRAF (4H58:C)	INSR (3ETA:A)
149	Y170	K600	I1157
150	S171	S601	Y1158
151	F172	R602	E1159
152	Q173	W603	T1160
153	M174	S604	D1161
154	A175	G605	Y1162
155	L176	S606	Y1163
156	-	H607	R1164
157	T177	E610	G1167
158	S178	Q611	K1168
159	V179	L612	G1169
160	V180	S613	L1170
161	V181	G614	L1171
162	T182	S615	P1172
163	L183	I616	V1173
164	W184	L617	R1174
165	Y185	W618	W1175
166	R186	M619	M1176
167	A187	A620	A1177
168	P188	P621	P1178
169	E189	E622	E1179
170	V190	V623	S1180
171	L191	I624	L1181
172	L192	R625	K1182
173	Q193	M626	D1183
174	S194	N630	G1184
175	S195	P631	V1185
176	Y196	Y632	F1186
177	A197	S633	T1187
178	T198	F634	T1188
179	P199	Q635	S1189
180	V200	S636	S1190
181	D201	D637	D1191
182	L202	V638	M1192
183	W203	Y639	W1193
184	S204	A640	S1194
185	V205	F641	F1195
186	G206	G642	G1196
187	C207	I643	V1197
188	I208	V644	V1198
189	F209	L645	L1199
190	A210	Y646	W1200

Alignment	CDK6 (1G3N:A)	BRAF (4H58:C)	INSR (3ETA:A)
191	E211	E647	E1201
192	M212	L648	I1202
193	F213	M649	T1203
194	R214	T650	S1204
195	R215	G651	L1205
196	K216	Q652	E1207
197	P217	L653	Q1208
198	L218	P654	P1209
199	F219	Y655	Y1210
200	R220	S656	Q1211
201	G221	N657	G1212
202	S222	N659	L1213
203	S223	N660	S1214
204	D224	R661	N1215
205	V225	D662	E1216
206	D226	Q663	Q1217
207	Q227	I664	V1218
208	L228	I665	L1219
209	G229	F666	K1220
210	K230	M667	F1221
211	I231	V668	V1222
212	L232	G669	M1223
213	D233	R670	D1224
214	V234	G671	G1225
215	I235	Y672	G1226
216	G236	L673	Y1227
217	L237	S674	L1228
218	P238	P675	D1229
219	G239	D676	Q1230
220	E240	L677	P1231
221	V266	R681	-
222	T267	S682	D1232
223	D268	N683	N1233
224	I269	C684	C1234
225	D270	P685	P1235
226	E271	K686	E1236
227	L272	A687	R1237
228	G273	M688	V1238
229	K274	K689	T1239
230	D275	R690	D1240
231	L276	L691	L1241
232	L277	M692	M1242

Alignment	CDK6 (1G3N:A)	BRAF (4H58:C)	INSR (3ETA:A)
233	L278	A693	R1243
234	K279	E694	M1244
235	C280	C695	C1245
236	L281	L696	W1246
237	T282	K697	Q1247
238	F283	K698	F1248
239	N284	K699	N1249
240	P285	R700	P1250
241	A286	D701	K1251
242	K287	E702	M1252
243	R288	R703	R1253
244	I289	P704	P1254
245	S290	L705	T1255
246	A291	F706	F1256
247	Y292	P707	L1257
248	S293	Q708	E1258
249	A294	I709	I1259
250	L295	L710	V1260
251	S296	A711	N1261
252	H297	S712	L1262
253	P298	I713	L1263
254	Y299	E714	K1264
255	F300	-	-
256	Q301	-	-
257	D302	-	-
258	-	-	-
259	-	-	-

Table S3. (*continued on next* page) 54 mutations totaling 108 FEP-calculated (alchemical) ΔGs in the active A and inactive basin B calculated from the BAR estimator, along with the corresponding errors which are default output from FEP+. Each row represents a single pair alchemical transformation in basins $\mathbb{A}$ and $\mathbb{B}$. $\Delta\Delta G$ is calculated from the difference $\Delta G(\mathbb{B}) - \Delta G(\mathbb{A})$, where positive differences indicate a shift in stability towards the active basin, and negative values indicate a shift towards the inactive basin. These mutations were identified from Potts $\Delta\Delta T$ scans, where a total of 18 double mutations located far in the left and right-hand tails of the histograms in Figure 3A were broken down into two single mutants each. The corresponding values of $\Delta\Delta G_{reorg}$ plotted in Figure 3C were calculated by averaging over three different alchemical paths connecting the double mutant with the constituent single mutants, resulting in 36 additional “intermediate” mutations to close the thermodynamic cycle and calculate an estimate of the error in $\Delta\Delta G_{reorg}$ due to hysteresis. The location of single-point mutations in the active or inactive-state tertiary structure are annotated in the “Structural Motifs” column.

	Mutation	ΔG(A)	BAR error	ΔG(B)	BAR error	ΔΔG	BAR error	ΔΔG (Avg)	Hysteresis error	ΔΔT Potts	Structural Motif
INSR	M53Y/R123K	7.98	±0.18	2.4	±0.14	-5.58	±0.23	-5.85	±0.58	-1.9	-
	M53Y	-0.13	±0.14	-3.76	±0.13	-3.63	±0.19	-3.88	±0.58	-0.94	R-spine
	R123K	9.08	±0.11	6.45	±0.07	-2.63	±0.13	-2.21	±0.58	-0.99	RD-pocket
	L115A/F143L	1.8	±0.12	-2.75	±0.13	-4.55	±0.18	-4.19	±0.43	-2.6	-
	L115A	-2.96	±0.08	-4.42	±0.19	-1.46	±0.21	-1.86	±0.43	-0.02	R-spine
	F143L	4.5	±0.10	1.63	±0.13	-2.87	±0.16	-2.9	±0.43	-0.49	R-spine
	F120I/F143Y	-1.37	±0.10	-5.11	±0.16	-3.74	±0.19	-3.36	±0.35	-2.1	-
	F120I	5.96	±0.08	5.84	±0.12	-0.12	±0.14	-0.29	±0.35	-1.01	N-term. anchor
	F143Y	-7.89	±0.08	-11.04	±0.12	-3.15	±0.14	-3.31	±0.35	-1	R-spine
	M145F/Y154L	20.07	±0.10	23.49	±0.17	3.42	±0.20	2.93	±0.45	1.9	-
	M145F	7.58	±0.10	8.16	±0.11	0.58	±0.15	0.67	±0.45	0.74	N-term. anchor
	Y154L	12.31	±0.08	15.26	±0.12	2.95	±0.14	3.26	±0.45	1.11	Substrate mimic
	R123L/Y154F	16.07	±0.14	18.81	±0.13	2.74	±0.19	2.85	±0.15	2.1	-
	R123L	4.94	±0.11	5.2	±0.08	0.26	±0.14	0.27	±0.15	0.42	RD-pocket
	Y154F	10.31	±0.11	13.12	±0.22	2.81	±0.25	2.65	±0.15	1.02	Substrate mimic
	R147A/Y154V	3.61	±0.07	11.28	±0.19	7.67	±0.20	6.57	±1.07	1.4	-
	R147A	-7.1	±0.05	-2.78	±0.19	4.32	±0.20	4.95	±1.07	0.36	RD-pocket
	Y154V	10.81	±0.07	13.82	±0.22	3.01	±0.23	3.58	±1.07	1.05	Substrate mimic

	Mutation	ΔG(A)	BAR error	ΔG(B)	BAR error	ΔΔG	BAR error	ΔΔG (Avg)	Hysteresis error	ΔΔT Potts	Structural Motif
BRAF	V52L/F143L	3.68	±0.10	4.5	±0.10	0.82	±0.14	1.19	±0.40	2.3	-
	V52L	0.8	±0.11	-0.16	±0.08	-0.96	±0.14	-0.97	±0.40	0.8	N-term. anchor
	F143L	3.77	±0.07	5.01	±0.11	1.24	±0.13	0.89	±0.40	0.33	R-spine
	F131V/F178E	-150.16	±0.11	-146.39	±0.09	3.77	±0.14	3.83	±0.55	2.4	-
	F131V	3.23	±0.09	6.56	±0.08	3.33	±0.12	2.83	±0.55	1.24	-
	F178E	-153.79	±0.07	-152.9	±0.06	0.89	±0.09	1.26	±0.55	1.11	αEF-αF loop
	F131A/E157T	23.79	±0.15	24.5	±0.12	0.71	±0.19	0.65	±0.06	2.7	-
	F131A	-2.35	±0.07	-1.2	±0.08	1.15	±0.11	1.18	±0.06	1.18	-
	E157T	26.33	±0.13	25.9	±0.10	-0.43	±0.16	-0.41	±0.06	1.53	-
	I120Y/L145Y	-29.56	±0.10	-28.89	±0.13	0.67	±0.16	0.77	±0.47	-2.7	-
	I120Y	-16.15	±0.09	-16.65	±0.10	-0.5	±0.13	-0.18	±0.47	-1.54	N-term. anchor
	L145Y	-13.53	±0.11	-12.4	±0.09	1.13	±0.14	0.71	±0.47	-1.19	N-term. anchor
	L53Y/I120R	-146.69	±0.14	-151.44	±0.15	-4.75	±0.21	-4.14	±0.53	-3.1	-
	L53Y	-12.44	±0.08	-11.98	±0.09	0.46	±0.12	0.35	±0.53	-1.34	R-spine
	I120R	-134.66	±0.09	-138.93	±0.09	-4.27	±0.13	-4.6	±0.53	-1.71	N-term. anchor
	L53F/A146T	11.99	±0.15	8.59	±0.16	-3.4	±0.22	-2.96	±0.90	-2.9	-
	L53F	-0.33	±0.09	-0.66	±0.12	-0.33	±0.15	0.18	±0.90	-1.17	R-spine
	A146T	12.28	±0.10	9.61	±0.08	-2.67	±0.13	-3.41	±0.90	-2.88	N-term. anchor

	Mutation	ΔG(A)	BAR error	ΔG(B)	BAR error	ΔΔG	BAR error	ΔΔG (Avg)	Hysteresis error	ΔΔT Potts	Structural Motif
CDK6	Y14L/T162Y	1.3	±0.13	8.04	±0.19	6.74	±0.23	6.5	±0.76	3.1	-
	Y14L	13.87	±0.07	15.18	±0.12	1.31	±0.13	1.9	±0.76	1.63	G-loop
	T162Y	-12.9	±0.11	-7.81	±0.14	5.09	±0.18	4.59	±0.76	1.55	C-term. anchor
	Y149L/V159F	11.16	±0.11	14.49	±0.12	3.33	±0.16	4.14	±0.89	3.1	-
	Y149L	11.78	±0.08	15.32	±0.06	3.54	±0.10	3.64	±0.89	2.29	N-term. anchor
	V159F	-0.74	±0.07	1.19	±0.08	1.93	±0.11	1.2	±0.89	0.67	RD-pocket
	R147N/L163K	-39.64	±0.16	-34.6	±0.10	5.05	±0.19	4.83	±0.38	2.5	-
	R147N	-42.48	±0.11	-41.08	±0.06	1.41	±0.12	1.18	±0.38	1.31	RD-pocket
	L163K	-130.41	±0.08	-127.15	±0.06	3.27	±0.10	3.59	±0.38	1.15	C-term. anchor
	V120L/V160F	2.76	±0.12	-1.37	±0.06	-4.13	±0.13	-4.84	±0.75	-5.2	-
	V120L	2.63	±0.08	-0.51	±0.05	-3.14	±0.10	-3.08	±0.75	-2.46	N-term. anchor
	V160F	0.57	±0.12	-1.13	±0.06	-1.7	±0.14	-1.02	±0.75	-2.77	RD-pocket
	I148D/V160R	-153.7	±0.19	-161.8	±0.05	-8.1	±0.20	-8.71	±3.07	-5.1	-
	I148D	-157.59	±0.06	-159.99	±0.02	-2.4	±0.06	-1.31	±3.07	-2.07	N-term. anchor
	V160R	-131.94	±0.34	-134.58	±0.04	-2.64	±0.34	-5.21	±3.07	-3.11	C-term. anchor
	R123K/Y176I	27.85	±0.17	23.65	±0.16	-4.2	±0.23	-4.29	±0.41	-6.3	-
	R123K	9.99	±0.12	5.44	±0.06	-4.55	±0.14	-4.24	±0.41	-4.09	RD-pocket
	Y176I	17.81	±0.17	17.29	±0.13	-0.52	±0.21	-0.84	±0.41	-2.3	αEF-αF loop
